# *Psychiatric Research and Clinical Practice:* The American Psychiatric Association's New Open‐Access Journal

**DOI:** 10.1176/appi.prcp.20180101

**Published:** 2019-04-19

**Authors:** Kimberly A. Yonkers

**Affiliations:** ^1^ Department of Psychiatry School of Epidemiology and Public Health Department of Obstetrics, Gynecology, and Reproductive Sciences Yale School of Medicine New Haven Conn.

Welcome to the first issue of the American Psychiatric Association's peer‐reviewed, open‐access journal, *Psychiatric Research and Clinical Practice (PRCP).* As the editor, it is my pleasure to share this premiere issue with our readers. The American Psychiatric Association is a leading publisher of research and information on mental health through its topflight journals and books. *PRCP* has a special niche in this collection—offering an open‐access journal confirms the Association's commitment to ensure that valuable information and research are available to all who may be interested. Providing open access is particularly critical as the general public shows a growing interest in psychiatry and neuroscience.

It is my honor to steward this new endeavor. I have a history of clinical research that spans over 20 years. I am fortunate to be joined by a talented editorial board that includes Drs. Renato Alarcón, Jonathan Alpert, Deanna Barch, Alex Kopelowicz, Teri Pearlstein, Katharine Phillips, Susan Schultz, and Lawson Wulsin. This board represents a variety of areas and interests and reflects the breadth of research we hope to see in the journal.

Readers and potential contributors may be interested in the sort of work we would like to receive and publish in *PRCP*. We welcome new research related to mental and behavioral health and particularly welcome submissions that move physicians closer to the bedside than to the bench. Works that are of high clinical relevance are a priority for us. We will also have a section titled “From the Lab,” which will provide important clinical context for laboratory findings. We are happy to consider replications of previous findings as well as research that finds different results. Qualitative and quantitative reviews that are helpful to our readers are appreciated. We look forward to receiving submissions that fall anywhere on the continuum from efficacy to effectiveness and even those focusing on implementation. Finally, “Clinical Practice Pearls” can facilitate our practice, and we would like to provide a platform for these bits of wisdom. As part of the family of journals from the American Psychiatric Association, the standards are understandably high, and readers should not expect less from the Association. We encourage those who are interested in publishing in *PRCP* to explore the guidance on our journal's Web site (prcp.psychiatryonline.org) and to contact us with any questions.

